# Double twist torsion testing to determine the non recrystallization temperature

**DOI:** 10.1038/s41598-021-81139-1

**Published:** 2021-01-15

**Authors:** Trevor J. Ballard, John G. Speer, Kip O. Findley, Emmanuel De Moor

**Affiliations:** Advanced Steel Processing and Products Research Center, Colorado School of Mines, 1500 Illinois St., Golden, CO 80401 USA

**Keywords:** Materials science, Structural materials, Metals and alloys

## Abstract

A double-twist torsion testing technique has been developed using a 316 stainless steel as an exemplar material to experimentally assess recrystallization behavior and determine the non-recrystallization temperature (T_nr_). This new method was compared to the traditional methods of double-hit compression and multi-step hot torsion testing. The double-twist torsion test allows T_nr_ to be related to the extent of austenite recrystallization through measurements of fractional softening while accommodating multiple deformation and recrystallization steps with a single specimen. The double-twist torsion test resulted in average T_nr_ values similar to those determined with multi-step hot torsion, and a partially recrystallized microstructure was observed in the vicinity of the calculated T_nr_ for all three methods. The ability of the double-twist torsion test to relate the experimental T_nr_ to the evolution of austenite recrystallization via fractional softening measurements while incorporating effects of multiple deformation steps offers an advantage over traditional methods for quantifying changes in austenite recrystallization during thermomechanical processing.

## Introduction

Effective simulation of thermomechanical processing at the laboratory scale requires techniques capable of linking the mechanical data generated during testing to the microstructural evolution of the material. Among the most common experimental techniques to simulate industrial hot rolling are double-hit compression and multi-step hot torsion testing^1,2^. Both techniques have been used to develop controlled rolling schedules for the production of microalloyed steels. Controlled rolling enables microstructural control of the material during thermomechanical processing as opposed to during subsequent heat treatments^3–5^. Design of effective controlled rolling schedules that achieve the desired microstructural control requires an understanding of the recrystallization behavior of the material being rolled. The recrystallization behavior is described by the non-recrystallization temperature (T_nr_), typically defined as the temperature below which complete static recrystallization ceases to occur during the interpass time between rolling passes, though slightly varying definitions exist^4,6,7^.

In a conventional controlled rolling schedule, roughing passes are conducted above T_nr_ where complete recrystallization occurs during the interpass time and refinement of the austenite microstructure occurs through multiple deformation and recrystallization cycles^1^. After the roughing passes, the material is cooled below T_nr_ and ideally into a temperature regime where recrystallization is fully suppressed to avoid extensive deformation in a temperature range where partial recrystallization of the austenite occurs^4,8^. Microstructural control and final dimensional tolerances of the product are achieved in microalloyed steels during finishing passes that take place in this temperature regime below T_nr_^1,4,7^. Because recrystallization is suppressed, strain is accumulated between finishing passes leading to an austenite microstructure consisting of elongated grains with increased grain boundary area, shear bands, and high dislocation density^9^. Upon cooling, these microstructural features serve as heterogeneous nucleation sites for ferrite, enhancing the ferrite nucleation rate and refining the final product microstructure^9^. Effective microstructural control, therefore, requires a reliable way to predict T_nr_ and changes in recrystallization behavior for a given alloy and process strategy. Both double-hit compression and multi-step hot torsion testing have been used to simulate industrial hot rolling and determine T_nr_. These two methods result in slightly different definitions, T_nr_ values, and corresponding microstructures depending on the method selected^7,10–13^.

Double-hit compression testing results in two true stress–true strain curves from which the amount of softening between hits is determined using the generalized equation:

1 $$ FS = \frac{{\sigma_{my} - \sigma_{ry} }}{{\sigma_{my} - \sigma_{oy} }} $$

where *FS* is the fractional softening, and *σ*_*my*_, *σ*_*ry*_, and *σ*_*oy*_ are the flow stresses corresponding to work hardened, partially recrystallized, and fully recrystallized material, respectively^14,15^. Different methods exist for quantifying the stresses in Eq. (1), some based on the stress at a given strain offset while others are based on the area under the true stress–true strain curves^15^. Some methods for determining the fractional softening, such as the 0.05 true strain method, have been found to provide results that correlate well with the recrystallized fraction^15,16^. The correlation between fractional softening and the recrystallized fraction establishes a direct link between the mechanical data generated during double-hit compression testing and microstructural changes. From the double-hit compression test, T_nr_ is defined as the temperature corresponding to a fractional softening of 0.2 as determined from Eq. (1)^2,17^. This definition of T_nr_ is based on the consideration that 20 pct of the total softening is typically attributed to recovery rather than recrystallization^18^. The transition from complete to incomplete recrystallization and, therefore, T_nr_, would thus occur at 20 pct softening based on this assumption. Unlike conditions in an industrial rolling mill, however, double-hit compression testing does not typically incorporate the cooling that occurs between consecutive deformation steps applied at progressively lower temperatures. Industrial processing also typically involves more deformation passes and higher pass strains than can be accommodated by a single compression specimen without excessive barreling. As a result, a single compression specimen is generally used to determine the extent of softening at a single temperature. Tests are conducted at a variety of temperatures to predict the evolution of microstructure and properties during a rolling schedule. The effects of multiple deformation events and recrystallization steps are, therefore, not incorporated.

To incorporate the effects of continuous cooling and consecutive deformation steps, multi-step hot torsion testing has been used as an alternative method to simulate thermomechanical processing at the laboratory scale^2,10,19–22^. Because torsion testing does not alter the specimen cross section, a larger number of deformation steps can be accommodated by a single specimen. Multi-step hot torsion testing involves consecutive deformation passes applied at progressively lower temperatures to a single specimen after first cooling from the soaking temperature. Thus, multi-step hot torsion testing allows the effects of accumulated strain and multiple deformation steps on changes in flow stress and observed microstructure to be captured. Multi-step hot torsion testing provides torque–twist curves for each deformation pass that are converted into equivalent stress–equivalent strain curves^10^. The mean flow stress (MFS) is determined for each pass and plotted as a function of the inverse absolute deformation temperature. T_nr_ is then identified as the temperature corresponding to a change in slope of the MFS versus inverse temperature relationship determined using a linear regression analysis. Unlike double-hit compression testing, deformation is not uniform through the cross section of a torsion specimen, and a direct correspondence between changes in flow stress and the extent of recrystallization at different locations within the cross section has not been studied extensively in the case of multi-step hot torsion testing^22,23^. While more representative of industrial processing than double-hit compression, multi-step hot torsion testing warrants additional attention to direct observation of the microstructure to fully characterize recrystallization behavior. Both methods make assumptions about the relationship between the mechanical and microstructural responses.

Homsher et al*.* compared T_nr_ for the two methods using six different microalloyed steels based on analysis of the force or torque^2^. Double-hit compression testing consistently predicted a higher T_nr_ than multi-step hot torsion testing. The difference between the results of the two methods was attributed to the effects of strain accumulation and grain size. In torsion testing, the application of consecutive deformation steps to a single specimen is believed to result in greater strain accumulation and a finer grain size near T_nr_ in comparison to double-hit compression testing where each specimen undergoes only a single deformation/recrystallization/deformation sequence. The increased dislocation density and grain boundary area in the multi-step hot torsion test are believed to promote recrystallization, thereby shifting T_nr_ to lower temperatures. In addition to the different T_nr_ values obtained for the same material, Homsher et al*.* confirmed that the prior austenite grain size measured at T_nr_ using double-hit compression was substantially larger than the prior austenite grain size at T_nr_ obtained from multi-step hot torsion testing.

For the current study, a *double-twist* torsion test to determine T_nr_ has been developed that involves pairs of isothermal deformation steps (separated by an interpass time) applied at consecutively lower temperatures to a single specimen. T_nr_ was determined both from measurements of fractional softening between isothermal deformation steps and from the MFS versus inverse temperature method using the series of first and second deformation steps at each temperature. The double-twist torsion test, therefore, allowed the effect of data analysis method, i.e. fractional softening and MFS versus inverse temperature, on T_nr_ to be assessed using results from a single test. T_nr_ determined using the double-twist torsion test was compared to T_nr_ determined via double-hit compression testing and multi-step hot torsion testing with similar deformation conditions and temperatures for all methods. A 316 austenitic stainless steel alloy was selected because phase transformations upon cooling, unlike the case of low carbon microalloyed steels, are absent and do not obscure the austenite microstructure, which can be directly assessed. The microstructures above, near, and below the calculated T_nr_ were also assessed for each method to determine how well the measured T_nr_ related to the definition of T_nr_ as the transition from complete to incomplete recrystallization during the interpass time between rolling passes. The evolution of grain size and grain morphology (strain accumulation) during thermomechanical processing was studied for each method and related to differences in T_nr_ determined from the traditional analysis of the stresses.

## Materials and methods

To better understand the relationship between changes in mechanical properties and the austenite microstructure, a commercial austenitic 316 stainless steel was chosen for analysis with composition given in Table 1. Martensite does not form upon quenching as in a microalloyed steel, and austenite is stable at both room temperature and the temperatures used for hot deformation enabling direct observation of the degree of recrystallization. In microalloyed steels, recrystallization is influenced by microalloy solutes, precipitates, or a combination thereof^20,24,25^. While 316 stainless steel does not form strong carbides or nitrides as a microalloyed steel does, the presence of molybdenum has been shown to reduce grain boundary mobility at high temperature and slow recrystallization^26^.

**Table 1:** Commercial 316 stainless steel bar composition.

wt pct	C	Mn	Si	Ni	Cr	Mo	N	S	P	Cu	Co
316	0.017	1.57	0.54	10.09	16.89	2.04	0.054	0.025	0.032	0.47	0.36

**Figure 1:**
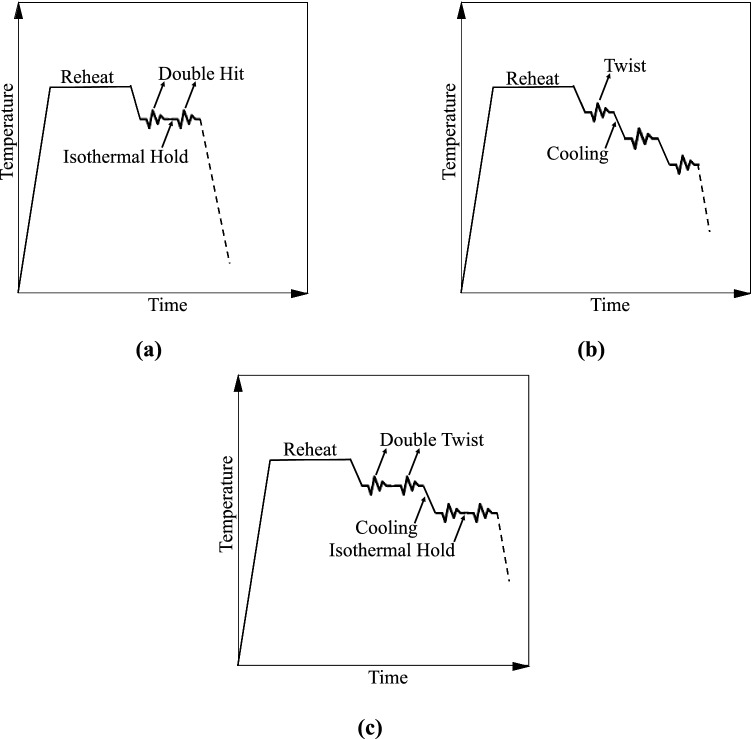
Schematic representations of thermomechanical simulation techniques of (**a**) double-hit compression, (**b**) multi-step hot torsion, and (**c**) double-twist torsion.

Bars of commercial 316 stainless steel with a 12.7 mm diameter were machined into sub-sized Gleeble torsion specimens with a gauge length of 14.4 mm and a gauge diameter of 7.2 mm^27^. Cylindrical Gleeble compression specimens with a diameter of 10.0 mm and a length of 15.0 mm were also machined^28^. Using the 316 stainless steel, T_nr_ was determined through processing on a Gleeble 3500 using both double-hit compression and multi-step hot torsion testing, and the microstructure was analyzed above, near, and below T_nr_ for each method. In addition, a double-twist torsion test was developed and conducted. Schematics of each testing method are given in Fig. 1.

### Double-hit compression testing

A schematic of the double-hit compression test is shown in Fig. 1a. Specimens were reheated to 1250 °C at a rate of 5 °C s^−1^, held for 1 min, and cooled to the selected deformation temperatures at a rate of 2.5 °C s^−1^. All specimens were deformed with a strain of 0.2 per hit at a strain rate of 0.1 s^−1^ with a 5 s interpass time between isothermal deformation steps. Specimens were air cooled to room temperature after the second deformation step. Double-hit compression tests were conducted at deformation temperatures of 1200–750 °C in 50 °C decrements. Because complete softening did not occur at 1200 °C, additional double-hit compression tests were conducted using 1250 and 1300 °C as both the reheating and deformation temperature to assess the temperature at which complete softening, and, therefore, complete recrystallization occurred. Material was available for only a single double-hit compression test at each temperature.

### Multi-step hot torsion testing

A schematic of the multi-step hot torsion test is shown in Fig. 1b. Specimens were reheated to 1250 °C at a rate of 5 °C s^−1^, held for one minute, and cooled to the first deformation temperature of 1200 °C at a rate of 2.5 °C s^−1^. A von Mises equivalent strain of 0.2 and a strain rate of 0.1 s^−1^ were used for all deformation steps. The specimen was cooled between deformation temperatures at a rate of 2.5 °C s^−1^. Consecutive deformation steps were applied at temperatures of 1200–750 °C in 50 °C decrements, and the specimen was air cooled to room temperature after the final deformation step. An average T_nr_ and 95 pct confidence interval was determined from the results of three multi-step hot torsion tests.

### Double-twist torsion testing

The double-twist torsion test utilizes the same deformation temperatures, deformation parameters, and number of deformation passes at each temperature as the double-hit compression test while allowing the effects of multiple deformation steps to be captured for a single specimen. A schematic of the test is shown in Fig. 1c. Specimens were reheated to 1250 °C at a rate of 5 °C s^−1^, held for 1 min, and cooled to the first deformation temperature of 1200 °C at a rate of 2.5 °C s^−1^. The specimen was then twisted to a von Mises equivalent strain of 0.2 at a strain rate of 0.1 s^−1^. The specimen was then held for 5 s at 1200 °C before being twisted again with the same parameters. After the second pass, the specimen was cooled at a rate of 2.5 °C s^−1^ to the next deformation temperature, and the double-twist process was repeated. Deformation passes were applied to a single specimen at temperatures of 1200–750 °C in 50 °C decrements, and the speciemen was air cooled to room temperature after the final deformation. Because complete softening did not occur at 1200 °C, an additional double-twist test was conducted using 1250 °C as both the reheating and deformation temperature for the purpose of determining the temperature at which complete softening, and, therefore, complete recrystallization, occurred. Two double-twist torsion tests were conducted to determine an average T_nr_ and 95 pct confidence interval.

### T_nr_ determination: fractional softening approach

T_nr_ was determined for both double-hit compression testing and double-twist torsion testing using calculations of the fractional softening that occurred during the interpass time between isothermal deformation steps. For the fractional softening approach, T_nr_ was taken as the temperature corresponding to a fractional softening of 0.2, or 20 pct. Several techniques are cited in the literature for obtaining fractional softening from the true stress–true strain data including the offset method^16,18,19^, the 0.05 true strain method^29^, the mean flow stress method^30^, and the back-extrapolation method^31^. For the present work, the 0.05 true strain method was selected to calculate fractional softening based on its extensive use in the literature and evidence suggesting that it correlates well with the recrystallized fraction^2,15–17,29^. Using the 0.05 true strain method, fractional softening at each temperature was calculated according to Eq. (2), given by:

2 $$ FS = \frac{{\sigma_{m,\;0.05\;TS} - \sigma_{2,\;0.05\;TS} }}{{\sigma_{m,\;0.05\;TS} - \sigma_{1,\;0.05\;TS} }} $$

where *σ*_*m, 0.05 TS*_ is the true stress at 0.05 true strain of a hypothetical second true stress–true strain curve corresponding to zero softening (i.e. an extrapolation of the first curve) and *σ*_1, 0.05* TS*_ and *σ*_2, 0.05* TS*_ are the true stresses at 0.05 true strain for the first and second deformation hits/passes, respectively^15,29^. The true stresses in Eq. (2) were determined from power law curves fitted to the experimental true stress–true strain data using the MATLAB curve fitting application. The *σ*_*m, 0.05 TS*_ term was determined from an extrapolation of the curve fit to the first true stress–true strain curve as the true stress corresponding to a true strain of 0.25. In addition to determining the *σ*_*m, 0.05 TS*_ term, curve fitting was helpful due to the high level of noise present in the compression data, especially at high temperature. This noise was attributed to current being passed through the sample to maintain the proper temperature during testing. A similar curve fitting procedure was applied to the double-twist torsion data. At higher strains, the data were not always optimally described by a power law fit. As a result, a rational curve fit was used in cases where a power law equation gave a poor fit. As in the case of double-hit compression, curve fits were performed using the MATLAB curve fitting application.

To determine T_nr_ (the temperature corresponding to 20 pct softening), fractional softening was plotted as a function of deformation temperature. Because T_nr_ did not always lie precisely at one of the tested deformation temperatures, further curve fitting was used to describe the fractional softening data. Fractional softening as a function of deformation temperature has been shown to follow a sigmoidal behavior in accordance with the assumption that fractional softening can be directly related to the recrystallized fraction^2^. Thus, a sigmoidal curve of the following form was applied using the MATLAB curve fitting application as a basis for describing the experimental fractional softening data:

3 $$ FS = A\left( {1 - \exp \left( { - B\left( {T - C} \right)^{n} } \right)} \right) + D $$

where *FS* is the fraction of softening, *T* is temperature in °C, and *A*, *B*, *C, n*, and *D* are scaling parameters adjusted until a fit of the data with an r^2^ value of at least 0.95 was obtained. Experimentally, it was observed that the fractional softening exceeded 100 pct at temperatures above 1200 °C. Fractional softening values in excess of 100 pct were thought to be the result of grain growth occurring after complete recrystallization thereby acting as an additional mechanism promoting softening beyond the starting value. Thus, fractional softening is only expected to correspond to the extent of recrystallization up to 100 pct softening. To define the upper shelf on the sigmoidal fit given by Eq. (3) and illustrate where complete recrystallization was expected, it was assumed that complete recrystallization occurred when softening values in excess of 100 pct were calculated.

### T_nr_ determination: mean flow stress (MFS) versus inverse temperature approach

T_nr_ was determined using the mean flow stress versus inverse temperature approach for the multi-step hot torsion test as well as the newly developed double-twist torsion test. For the double-twist torsion test, the MFS versus inverse temperature approach was applied (separately) to the series of first deformation passes and the series of second deformation passes at every temperature, treating each series as a separate multi-step hot torsion test.

Hot torsion testing on the Gleeble 3500 resulted in a series of torque–twist curves, one for each deformation pass in the test. The torque–twist data recorded during testing were converted into equivalent stress–equivalent strain data. Equivalent stress for each pass was calculated according to:

4 $$ \overline{\sigma } = \frac{3\sqrt 3 T}{{2\pi a^{3} }} $$

where $\sigma$ is the equivalent stress, *T* is torque, and *a* is the radius of the gauge section^22,32^. Equivalent strain was calculated using the equation:

5 $$ \overline{\varepsilon } = \frac{0.724a\theta }{{\sqrt 3 L}} $$

where $\stackrel{-}{\varepsilon }$ is equivalent strain, *a* is the radius of the gauge section, $\theta$ is the twist angle in radians, and *L* is the length of the gauge section^22,32^. Note that Eq. (5) is multiplied by 0.724 to represent strain at the “effective” radius, where effects of the structural gradient and variance in strain rate sensitivity and strain hardening behavior through the cross-section of the torsion specimen are considered to be minimized^22^. Using the equivalent stress–equivalent strain data, MFS was calculated for every pass according to:

6 $$ MFS = \frac{1}{{\overline{{\varepsilon_{b} }} - \overline{{\varepsilon_{a} }} }}\int\limits_{{\overline{{\varepsilon_{a} }} }}^{{\overline{{\varepsilon_{b} }} }} {\overline{\sigma } \;d\overline{\varepsilon } } $$

where *MFS* is the mean flow stress, $\stackrel{-}{{\varepsilon }_{a}}$ and $\stackrel{-}{{\varepsilon }_{b}}$ are the initial and final equivalent strains per pass, respectively, and $\bar{\sigma }$ is the equivalent stress per pass^10^. MFS was then plotted as a function of the inverse absolute deformation temperature, resulting in two linear regions^10^. The temperature at the intersection of the linear regions was defined as T_nr_ for the torsion test. The integral in Eq. (6) was evaluated in MATLAB using a trapezoid approximation.

The data included in the two separate linear regions were identified using a statistical method outlined by Homsher et al*.* to minimize uncertainty in determining T_nr_ from MFS data^33^. For the set of MFS data obtained from a multi-step hot torsion test, a line was fitted to the first three data points, which were treated as region I. The fourth point was skipped, and a second line was fitted to the remaining data points, which were treated as region II. The r^2^ value, a statistical measurement of variance, was determined for each line, and the two values were multiplied together. The process was repeated by fitting a line to the first four data points, skipping the fifth data point, and fitting a second line to the remaining data points. The r^2^ values were again determined for each region and multiplied together. This process was continued until there were only three data points in the second region. The pair of lines with the greatest product of r^2^ values was treated as the appropriate fit to regions I and II. The temperature corresponding to the intersection of the two lines with the greatest product of r^2^ values was taken as the experimentally determined T_nr_^33^.

### Microstructural analysis

The austenite microstructure was studied above, near, and below the experimentally determined T_nr_ for all testing methods using light optical microscopy. A testing temperature near the experimental T_nr_ was selected for microstructural analysis with additional microstructures being analyzed at testing temperatures 50–100 °C above and below T_nr_. Interrupted torsion tests were conducted by deforming specimens until the temperature of interest above, near, or below T_nr_ was reached and air cooling to room temperature. Quenching was not deemed necessary as phase transformations that obscure the austenite microstructure do not occur in the 316 stainless steel being studied.

Compression specimens were sectioned parallel to the compression axis. For torsion specimens, the gauge section was removed and ground longitudinally on the tangential plane to the effective radius of 72.4 pct of the specimen radius as determined from measurements of chord length following the procedure of Whitely et al.^22,23^. Specimens were mounted in Bakelite and polished to a 1 µm finish using standard metallographic procedures. The microstructures were evaluated in the longitudinal direction near the center of the specimens. All specimens were electrochemically etched in a solution of 60 pct nitric acid in water with a potential of 1 V and a current of approximately 0.15 A.

The qualitative study of the microstructures above, near, and below T_nr_ allowed the ability of each method to produce a microstructure consistent with the definition of T_nr_ as the transition from complete to incomplete recrystallization to be assessed. Because differences in T_nr_ predicted between double-hit compression and multi-step hot torsion testing have been attributed to differences in grain size and strain accumulation, the changes in average grain size and grain aspect ratio were also measured and compared between test methods at various stages during thermomechanical processing. The average grain size was determined using a concentric three-circle intercept procedure with a minimum of 500 intercepts counted for each condition to ensure statistical validity. To measure grain aspect ratio, ImageJ image processing software was used. Microstructures were converted into binary images with grain boundaries outlined in white surrounding black grain interiors. Noise and effects from manganese sulfide (MnS) inclusions were removed using the ‘de-speckle’ function in ImageJ. An ellipse was then fitted to each grain, and the aspect ratio was manually determined by dividing the long and short dimensions of the ellipse. The aspect ratios of a minimum of 100 grains above, near, and below T_nr_ for each method were measured. The aspect ratio distribution parallel to the rolling direction in the as-received condition was also determined. The microstructure was equiaxed, and the distribution in aspect ratios above, near, and below T_nr_ was compared to this equiaxed condition. Comparison of average grain size and changes in aspect ratio allowed for the effect of the various imposed thermomechanical histories on T_nr_ and the resulting microstructure to be assessed. Finally, Vickers microhardness was used to assess the effect of each processing method on changes in mechanical properties above, near, and below T_nr_. Ten indents using a 0.5 kg load and a 10 s dwell time were performed to determine an average hardness and 95 pct confidence interval.

## Results and discussion

Gleeble 3500 data for double-hit compression, multi-step hot torsion, and double-twist torsion testing on 316 stainless steel are presented and discussed. Differences in the mechanically determined T_nr_ are related to differences in average grain size and aspect ratio between the three methods assessed for T_nr_ determination. Vickers microhardness measurements on specimens deformed above, near, and below T_nr_ are related to the microstructural evolution in each testing method and show the progressive changes in mechanical properties during processing.

### T_nr_ determination

**Figure 2:**
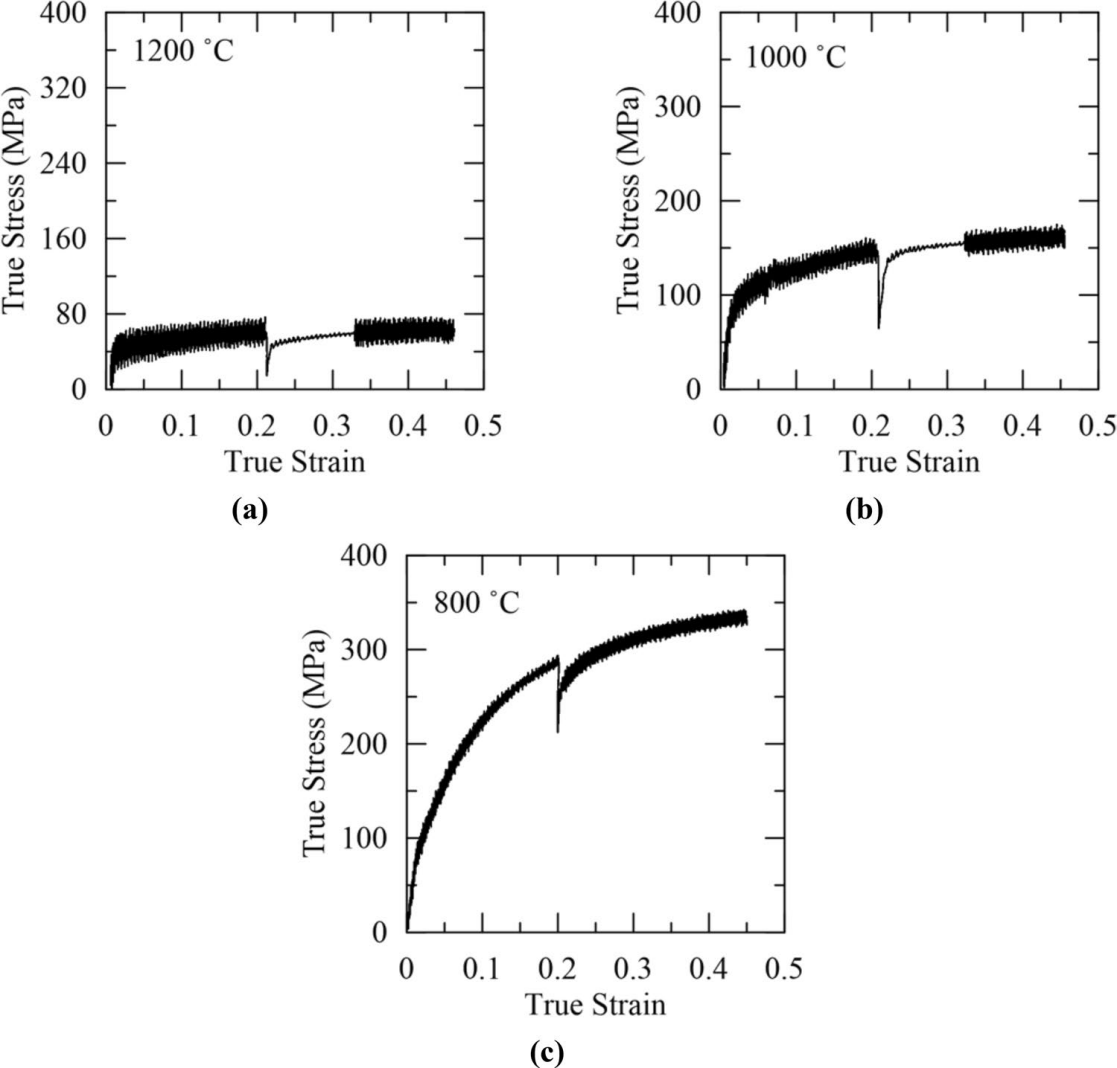
Representative true stress–true strain curves from Gleeble 3500 double-hit compression testing at temperatures of (**a**) 1200 °C, (**b**) 1000 °C, and (**c**) 800 °C.

Representative sets of the true stress–true strain curves used to determine fractional softening from the double-hit compression test for temperatures of 1200 °C, 1100 °C and 1000 °C are shown in Fig. 2a–c, respectively to illustrate changes in mechanical behavior at different temperatures. An increase in flow stress was clearly observed as the deformation temperature was reduced. Figure 2 also illustrates the high level of noise present in the compression data, especially at temperatures above 1000 °C. The noise in the data was related to the heating pulses applied during resistive heating on the Gleeble 3500. One method to mitigate such noise is to avoid heating *during* deformation. To confirm that the noise in the data was due to heating, sample tests were conducted without heating during deformation, and the amount of noise in the data substantially decreased, indicating that the oscillations in the true stress–true strain curves shown in Fig. 2 could be treated as noise. However, the time required to maintain a strain rate of 0.1 s^−1^ led to a temperature drop during deformation when no heating was applied. Thus, the resistive heating capabilities of the Gleeble 3500 were enabled during testing to ensure the desired deformation temperature was maintained, and the curve fitting procedure previously detailed was used for analysis of the true stress–true strain data. Comparing the curves in Fig. 2, it was evident that the noise decreased at lower temperature. The decrease in noise was attributed to a lower current being required to maintain the proper testing temperature.

**Figure 3:**
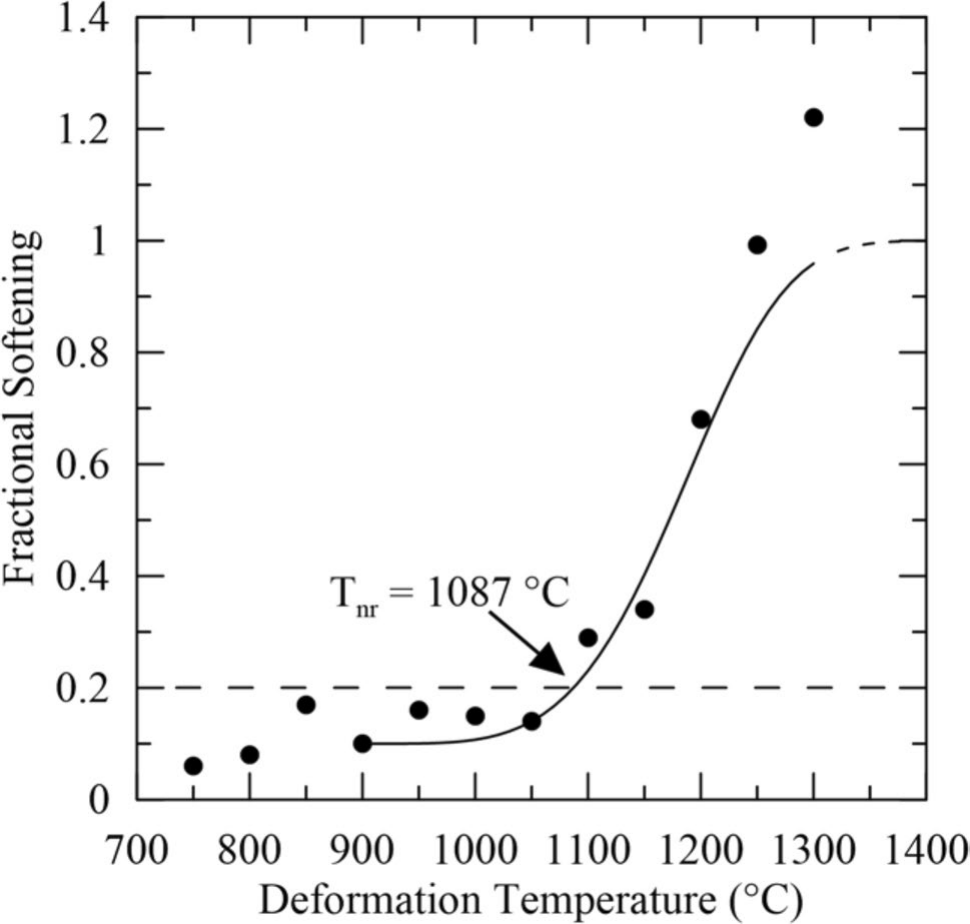
Fractional softening versus deformation temperature for Gleeble 3500 double-hit compression testing. The calculated T_nr_ is 1087 °C using the assumption that T_nr_ corresponds to 20 pct fractional softening. The dashed region at the top of the fitted sigmoidal curve shows where complete recrystallization was inferred as softening values in excess of 100 pct were obtained.

Fractional softening was plotted against deformation temperature, and the results are shown in Fig. 3 along with the curve fitted to the data. A reduction in fractional softening with decreasing temperature was observed, as expected. However, an “upper shelf” for the extent of softening was not observed at the temperatures tested and complete sigmoidal behavior was not evident, unlike results reported for some microalloyed steels^2,34^. The dashed portion at the top of the fitted curve in Fig. 3 shows where the upper shelf for fractional softening and complete recrystallization was expected to lie assuming complete recrystallization when fractional softening exceeded 100 pct. The fractional softening values in excess of 100 pct presumably indicate that additional softening mechanisms, such as grain growth, were active. Thus, the fractional softening data were only expected to correlate with the extent of recrystallization up to a softening of 100 pct. T_nr_ was determined to be 1087 °C using the assumption that T_nr_ corresponds to 20 pct fractional softening.

**Figure 4:**
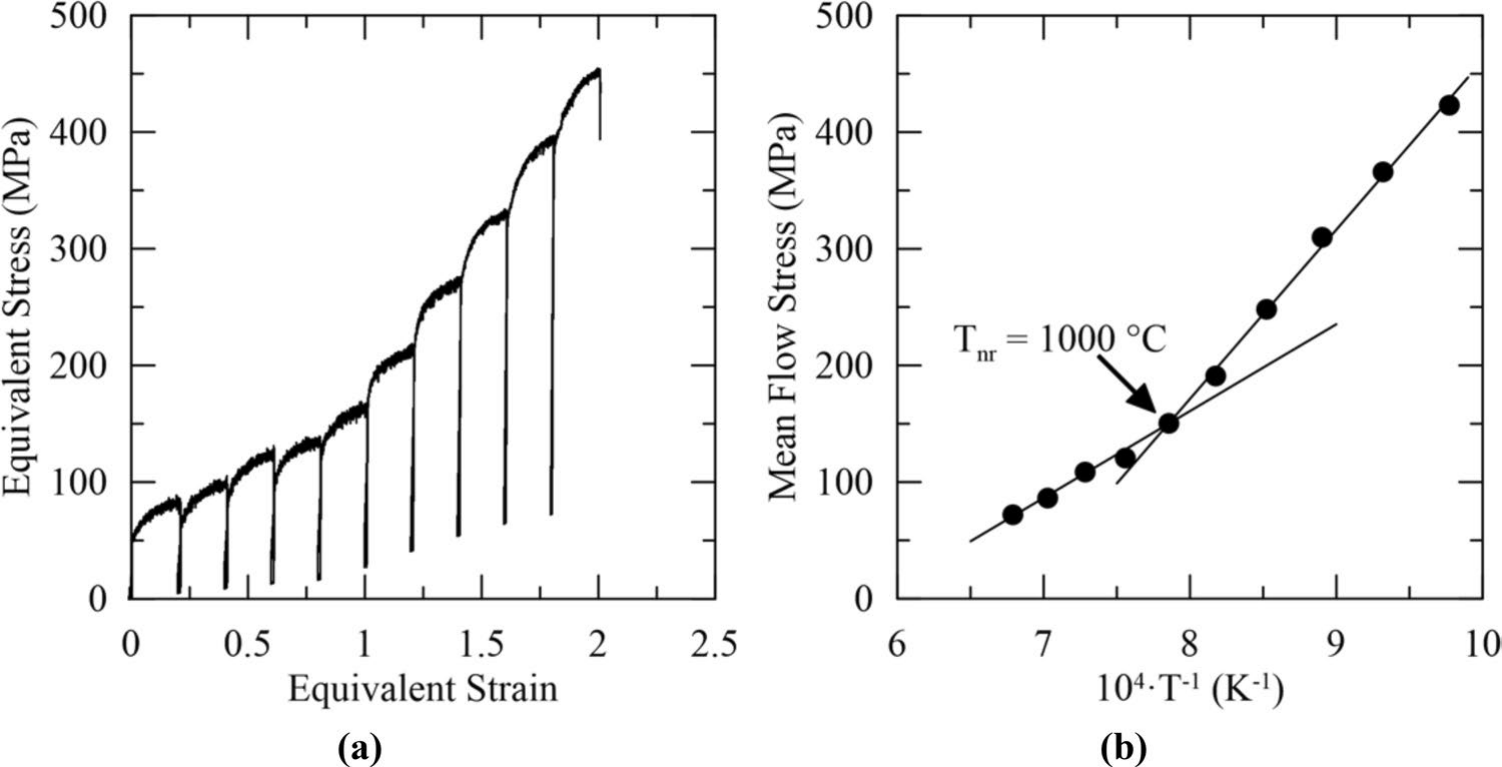
Mechanical data from a ten pass multi-step hot torsion test showing the (**a**) equivalent stress–equivalent strain curves and (**b**) MFS versus inverse temperature results with T_nr_ determined to be 1000 °C for this trial.

A representative series of equivalent stress–equivalent strain curves and the resulting MFS versus inverse temperature data from the 10-pass multi-step hot torsion test are shown in Fig. 4a,b, respectively. Two distinct linear regions were evident in Fig. 4b, and T_nr_ was determined to be 1000 °C at their intersection. From two additional multi-step hot torsion tests, the average T_nr_ from multi-step hot torsion testing was determined to be 1037 ± 31 °C. In microalloyed steels, precipitation occurs at lower temperatures and impedes the motion of grain boundaries, thereby preventing recrystallization and allowing accumulation of strain between rolling passes^1^. In the 316 stainless steel, molybdenum can act via a solute drag mechanism to impede grain boundary motion and elevate the temperature of the distinct inflection point shown in Fig. 4b^26^. The mechanically determined T_nr_ was substantially lower when determined from the multi-step hot torsion test compared to the double-hit compression test, consistent with trends observed in literature for microalloyed steels^2^.

**Figure 5:**
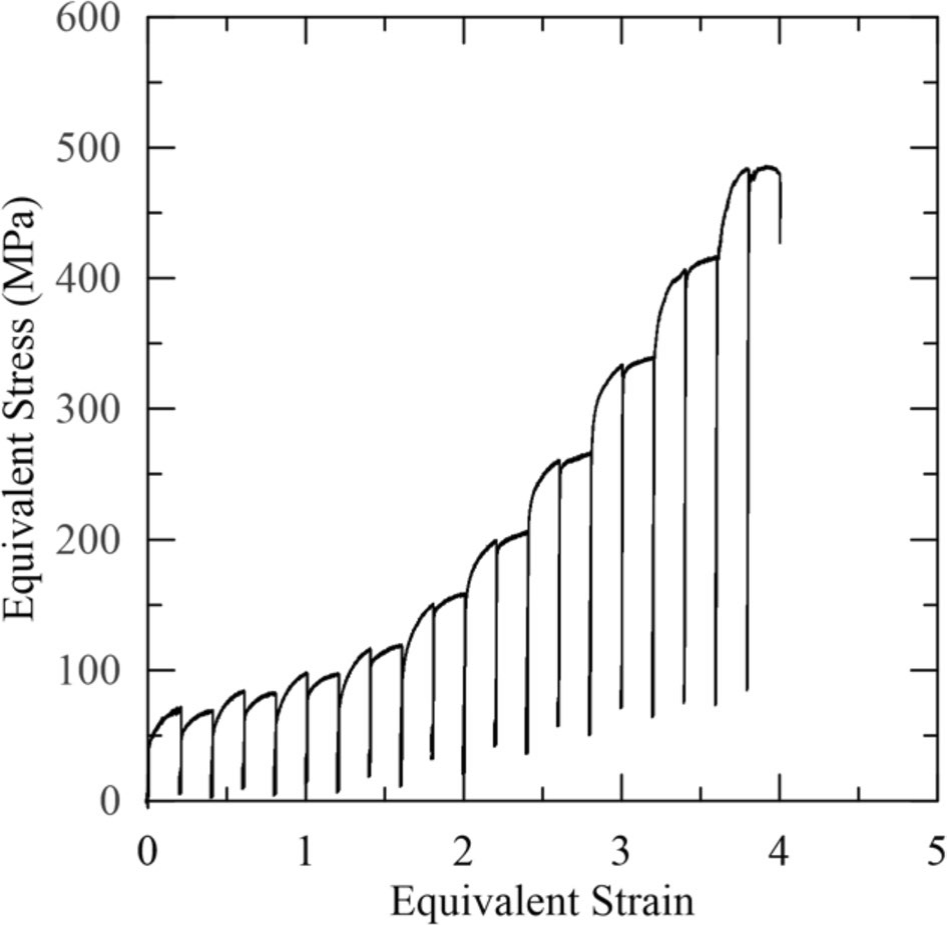
Equivalent stress–equivalent strain curves from Gleeble 3500 double-twist torsion testing.

**Figure 6:**
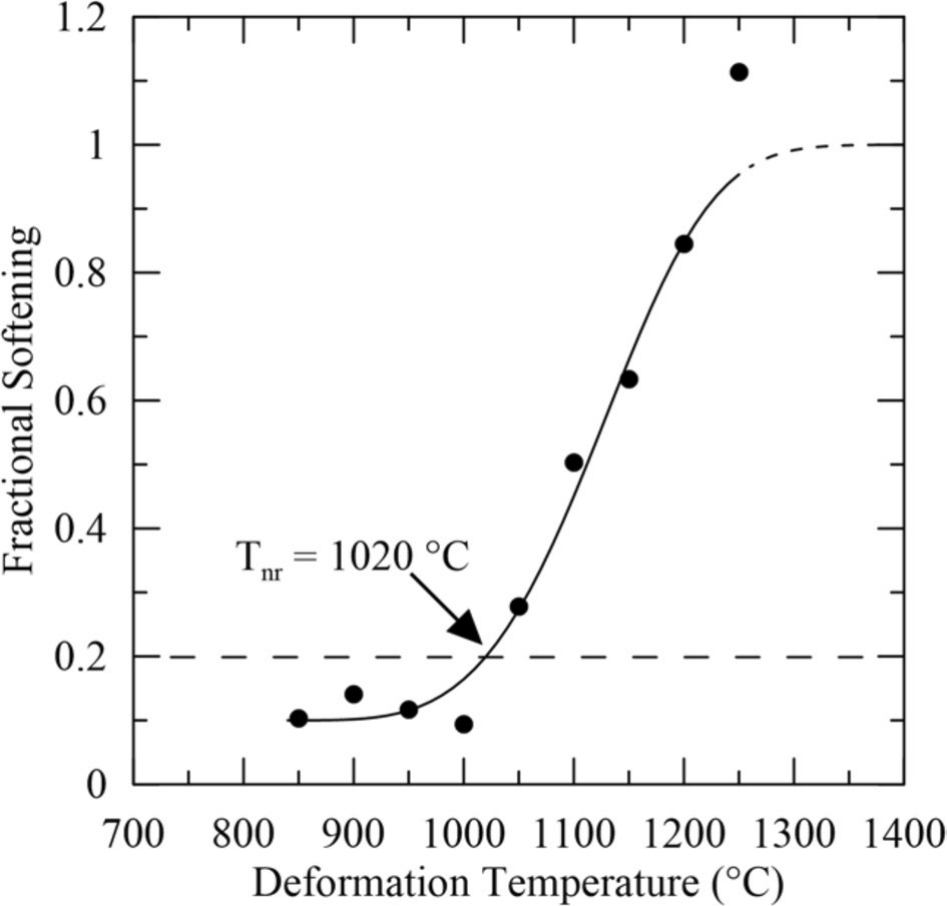
Fractional softening versus deformation temperature for double-twist torsion testing. T_nr_ was determined to be 1020 °C for this trial. The dashed region on the sigmoidal curve shows where complete recrystallization was inferred as fractional softening values exceeded 100 pct.

From the double-twist torsion test, T_nr_ was determined using both the fractional softening approach and the MFS versus inverse temperature approach, treating the series of first and second deformation passes at each temperature as separate multi-step hot torsion tests. A representative series of equivalent stress–equivalent strain curves from the double-twist torsion test used in the fractional softening and mean flow stress calculations are shown in Fig. 5. Fractional softening as a function of deformation temperature is shown in Fig. 6 along with the curve used to model the fractional softening data. Note that at 800 °C, a negative value of fractional softening was reported while at 750 °C, the specimen appeared to fracture near the end of the second twist. Consequently, data from these two temperatures were excluded from the curve fit presented in Fig. 6, and T_nr_ was calculated to be 1020 °C. From the two double-twist torsion tests, T_nr_ was determined to be 1014 ± 9 °C.

Similar to the double-hit compression tests that were conducted, an upper-shelf was not distinctly observed in the fractional softening data as would be represented by sigmoidal behavior. At 1250 °C, the fractional softening was 1.11 indicating that complete recrystallization had occurred. The upper shelf on fractional softening and complete recrystallization, therefore, can be inferred to lie between 1200 and 1250 °C shown by the dashed portion of the fitted curve in Fig. 6. The upper shelf was slightly lower than for double-hit compression, where complete softening and, therefore, complete recrystallization, were inferred to lie at temperatures slightly greater than 1250 °C, where there was 99 pct softening.

**Figure 7:**
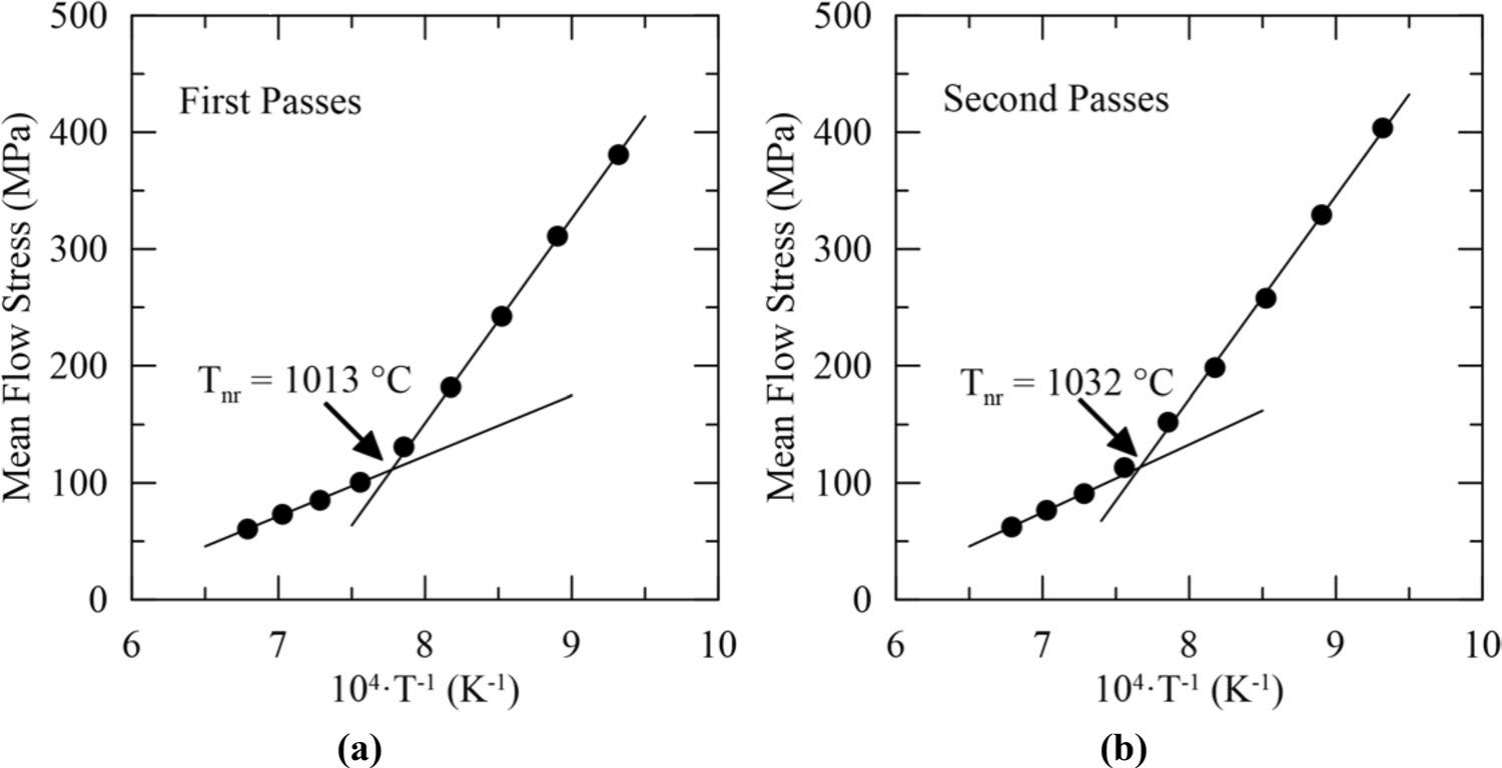
T_nr_ determination from the double-twist torsion test using the MFS versus inverse temperature approach for the set of (**a**) first and (**b**) second deformation passes. T_nr_ was determined to be 1013 and 1032 °C from the series of first and second deformation passes, respectively, for this trial.

T_nr_ was determined using the MFS versus inverse temperature approach for the series of first and second passes at each temperature from two replicates. A representative series of MFS versus inverse absolute deformation temperature curves used to determine T_nr_ from the series of first and second passes in the double-twist test is given in Fig. 7a,b, respectively. T_nr_ was determined to be 1013 °C and 1032 °C from the set of first and second deformation passes, respectively. From the two replicates, average T_nr_ values of 1030 ± 23 and 1041 ± 13 °C were determined from the series of first and second deformation passes, respectively. Both average T_nr_ values are within the same 50 °C temperature decrement indicating good correspondence between the two T_nr_ values for the 316 stainless steel used in the present study.

Table 2 summarizes the calculated T_nr_ values for each method used. The double-twist torsion test resulted in average T_nr_ values similar to those determined with multi-step hot torsion testing. T_nr_ from the fractional softening approach (1014 ± 9 °C) was near, though slightly lower than, T_nr_ calculated from the first (1030 ± 23 °C) and second (1041 ± 13 °C) set of deformation passes, respectively using the mean flow stress versus inverse temperature approach. When analyzed as a multi-step hot torsion test with the mean flow stress versus inverse temperature approach, T_nr_ in the double-twist method was close to that calculated from the conventional (single-twist) multi-step hot torsion test.

**Table 2:** T_nr_ for 316 stainless steel determined by different testing methods.

Method	T_nr_ (°C)
Double-hit compression	1087^a^
Double-twist torsion (fractional softening)	1014 ± 9
Double-twist torsion (first pass)	1030 ± 23
Double-twist torsion (second pass)	1041 ± 13
Multi-step hot torsion	1037 ± 31

^a^Material was available for only a single test per temperature. No uncertainty value is available.

Various authors have studied torsion testing using austenitic stainless steels^26,35,36^. The T_nr_ values reported in Table 2 are generally consistent with values found in literature for similar alloys. Ryan et al. found T_nr_ to be approximately 1000 °C using multi-step hot torsion for a 316 stainless steel with a strain rate of 0.1 s^−1^^26^. Other processing parameters including the pass strain and interpass time were not the same as those used in the current study making direct comparison difficult. Giordani et al. studied multi-step hot torsion testing on a 0.42Nb–0.37N (wt pct) austenitic stainless steel and found T_nr_ to be approximately 1110 °C, higher than reported in the current study possibly due to the Nb alloying content^35^. The previously described work by Homsher et al*.* using six different low carbon microalloyed plate steels found a difference of up to 71 °C in T_nr_ between double-hit compression and multi-step hot torsion testing^2^. The current study, which utilized ten passes in both double-hit compression and multi-step hot torsion testing, found a difference of 50 °C in average T_nr_ between the two methods, similar to the trend reported by Homsher et al.

When comparing the double-twist test data analyzed with fractional softening or MFS versus inverse temperature, better agreement was observed in T_nr_ values determined from the two data analysis methods than when comparing T_nr_ predicted with double-hit compression to multi-step hot torsion testing. All T_nr_ values from the double-twist torsion test were within the same 50 °C temperature decrement, indicating good correspondence between the values. The general proximity of the T_nr_ values indicates that the method of data analysis, i.e*.* fractional softening or MFS versus inverse temperature, has only a modest effect on the calculated T_nr_ when processing conditions are held constant for the 316 stainless steel. The origin of the slight differences between the T_nr_ values in the double-twist torsion test may be the result of the relatively large 50 °C temperature decrements used in the current study. Smaller temperature decrements may result in T_nr_ values with better agreement. Furthermore, the linear fits used in the MFS versus inverse temperature approach are sensitive to small changes in mean flow stress between passes and may also contribute to the differences observed between the T_nr_ values calculated from the double-twist torsion test.

### Microstructural analysis

**Figure 8:**
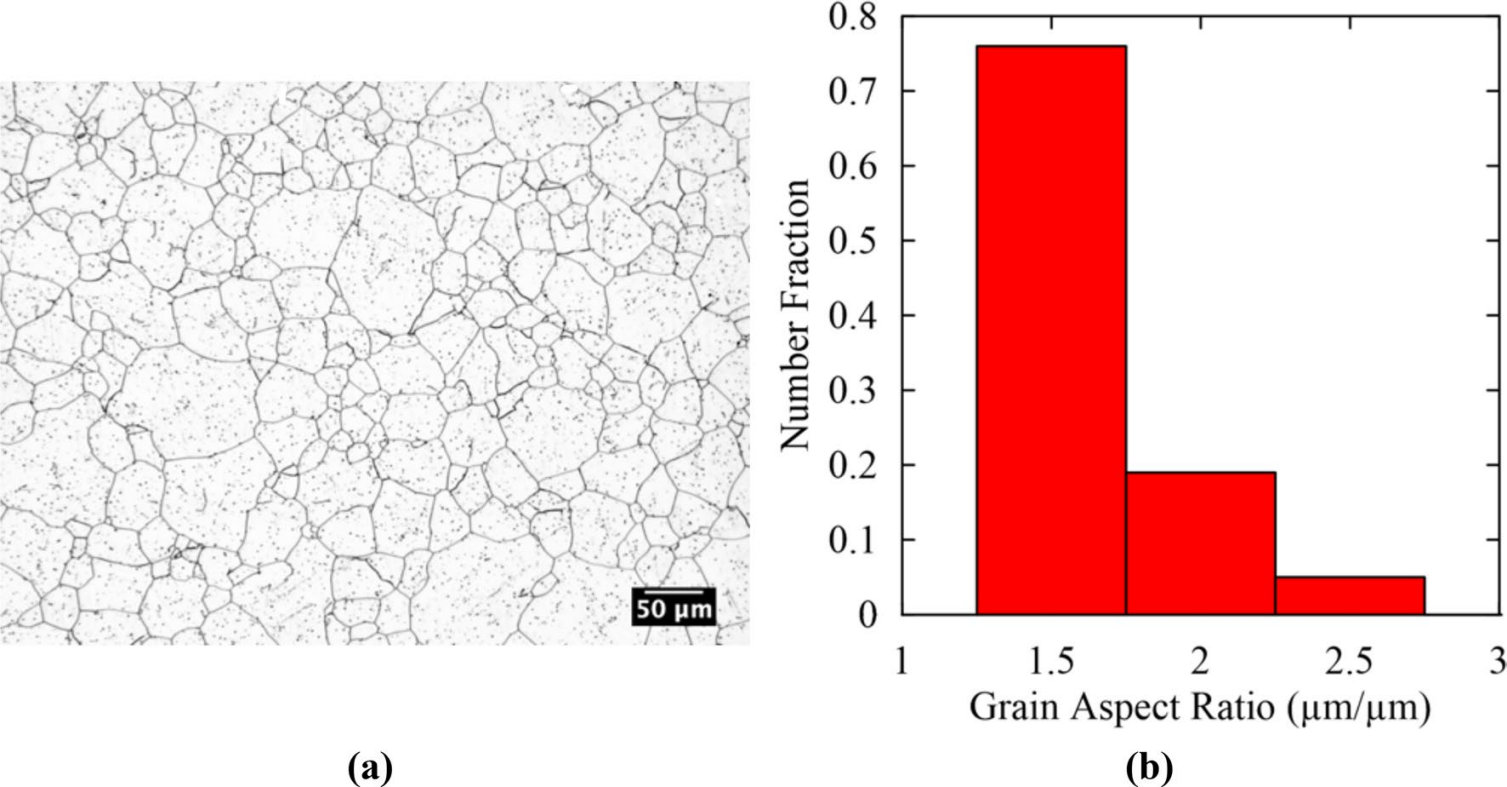
(**a**) As-received, equiaxed microstructure parallel to the rolling direction and (**b**) grain aspect ratio histogram for the microstructure in (**a**).

Microstructures above, near, and below the experimentally measured T_nr_ were assessed for each method. The equiaxed as-received microstructure was also analyzed parallel to the rolling direction. The as-received microstructure is shown in Fig. 8a along with the distribution in aspect ratios in Fig. 8b. Given that a fraction of 0.95 grains in the microstructure in Fig. 8a had aspect ratios below 2.0, the current study defines grains with aspect ratios greater than 2.0 as deformed.

**Figure 9:**
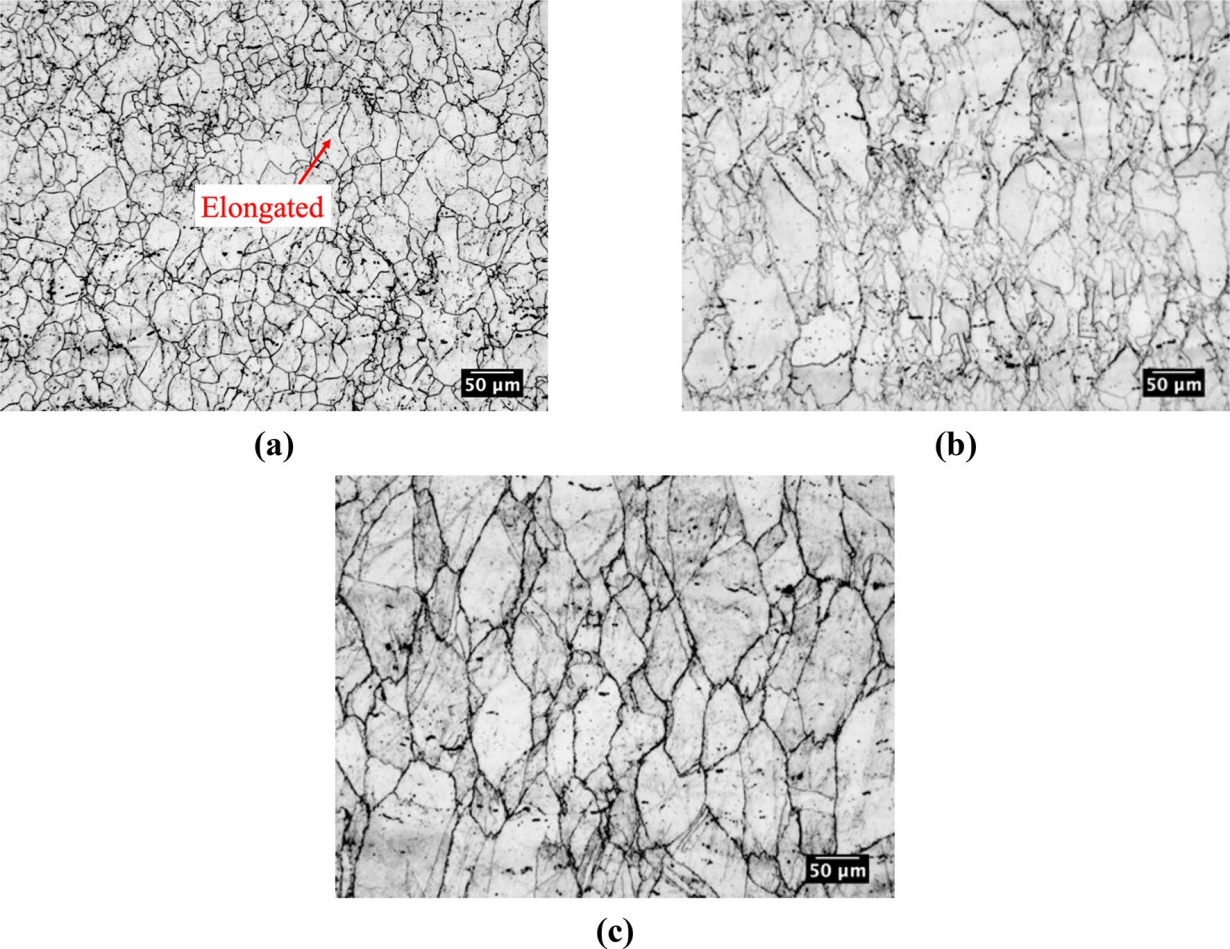
Microstructures generated using double-hit compression with the compression axis horizontal to the page (**a**) above T_nr_ at 1150 °C, (**b**) near T_nr_ at 1100 °C, and (**c**) below T_nr_ at 1050 °C. Specimens were electrochemically etched in a 60 pct nitric acid and 40 pct water solution.

For double-hit compression testing, the microstructures above T_nr_ at 1150 °C, near T_nr_ at 1100 °C, and below T_nr_ at 1050 °C are shown in Fig. 9a–c, respectively. Above T_nr_ at 1150 °C, the microstructure was mostly equiaxed, suggesting a predominance of recrystallized grains. However, some elongated grains, as identified in Fig. 9a, were also present, possibly indicating some strain accumulation above the measured T_nr_. Strain accumulation above T_nr_ was confirmed by measurements of grain aspect ratio presented later in this section. Near T_nr_ at 1100 °C, large elongated grains were surrounded by smaller equiaxed grains suggestive of partial recrystallization near T_nr_. Below T_nr_ at 1050 °C, a microstructure with coarse elongated grains was observed, characteristic of a microstructure below T_nr_. Thus, a dramatic change in microstructure occurred over the 100 °C range applicable to Fig. 9.

The average grain size was determined for the same deformation temperatures as in Fig. 9. The average grain size increased with decreasing deformation temperature. Above the measured T_nr_, the average grain size was approximately 25 µm. Near T_nr_ at 1100 °C, the average grain size increased to approximately 30 µm. A further increase in average grain size to approximately 37 µm was observed below T_nr_ at 1050 °C. The increase in grain size with decreasing deformation temperature was attributed to recrystallization at high deformation temperatures, perhaps along with some grain growth as the specimen cooled from the soaking temperature of 1250 °C to the deformation temperature.

**Figure 10:**
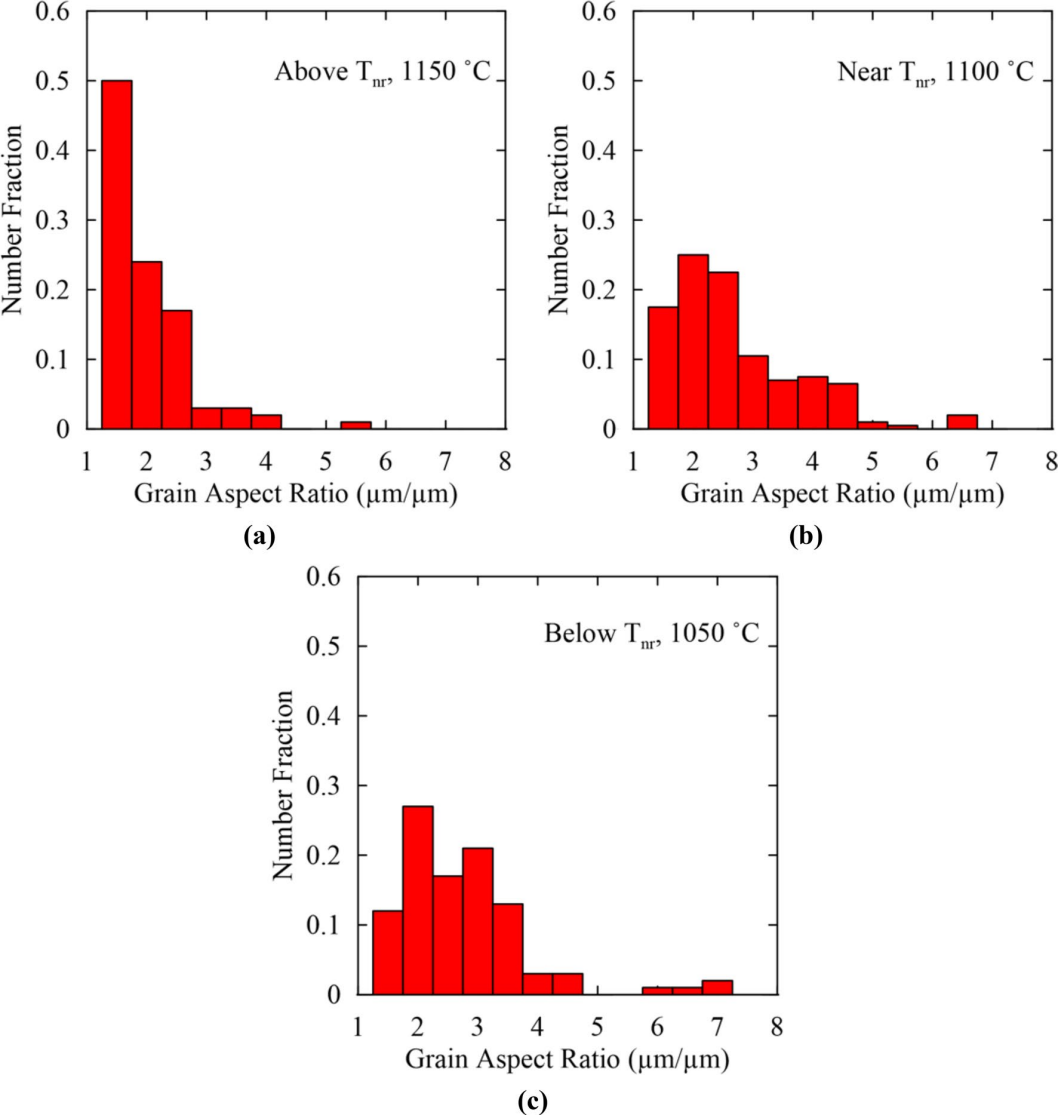
Grain aspect ratio histograms for double-hit compression testing at temperatures (**a**) above T_nr_ at 1150 °C, (**b**) near T_nr_ at 1100 °C, and (**c**) below T_nr_ at 1050 °C.

The change in strain accumulation was studied using measurements of grain aspect ratio above T_nr_ at 1150 °C, near T_nr_ at 1100 °C, and below T_nr_ at 1050 °C and is shown in Fig. 10a–c, respectively. Based on the aspect ratio distribution in Fig. 8, grains with aspect ratios greater than 2.0 were considered to be deformed while grains with lower aspect ratios were considered recrystallized. Above T_nr_, a fraction of 0.74 of the measured grains had aspect ratios below 2.0 indicating minimal strain accumulation and a mostly recrystallized microstructure. However, the fraction of remaining grains showed higher aspect ratios suggesting some strain accumulation above T_nr_ and thereby supporting the fractional softening data which indicated incomplete softening and, therefore, incomplete recrystallization above the measured T_nr_. Near T_nr_, grain aspect ratios increased further. A fraction of 0.43 grains had aspect ratios between 1.0 and 2.0 while the remaining grains had higher aspect ratios. Thus, in the vicinity of T_nr_ a mixed microstructure with both recrystallized and deformed grains was present. Below T_nr_ at 1050 °C, a fraction of 0.39 grains had aspect ratios between 1.0 and 2.0 indicating greater strain accumulation than at the higher temperatures.

**Figure 11:**
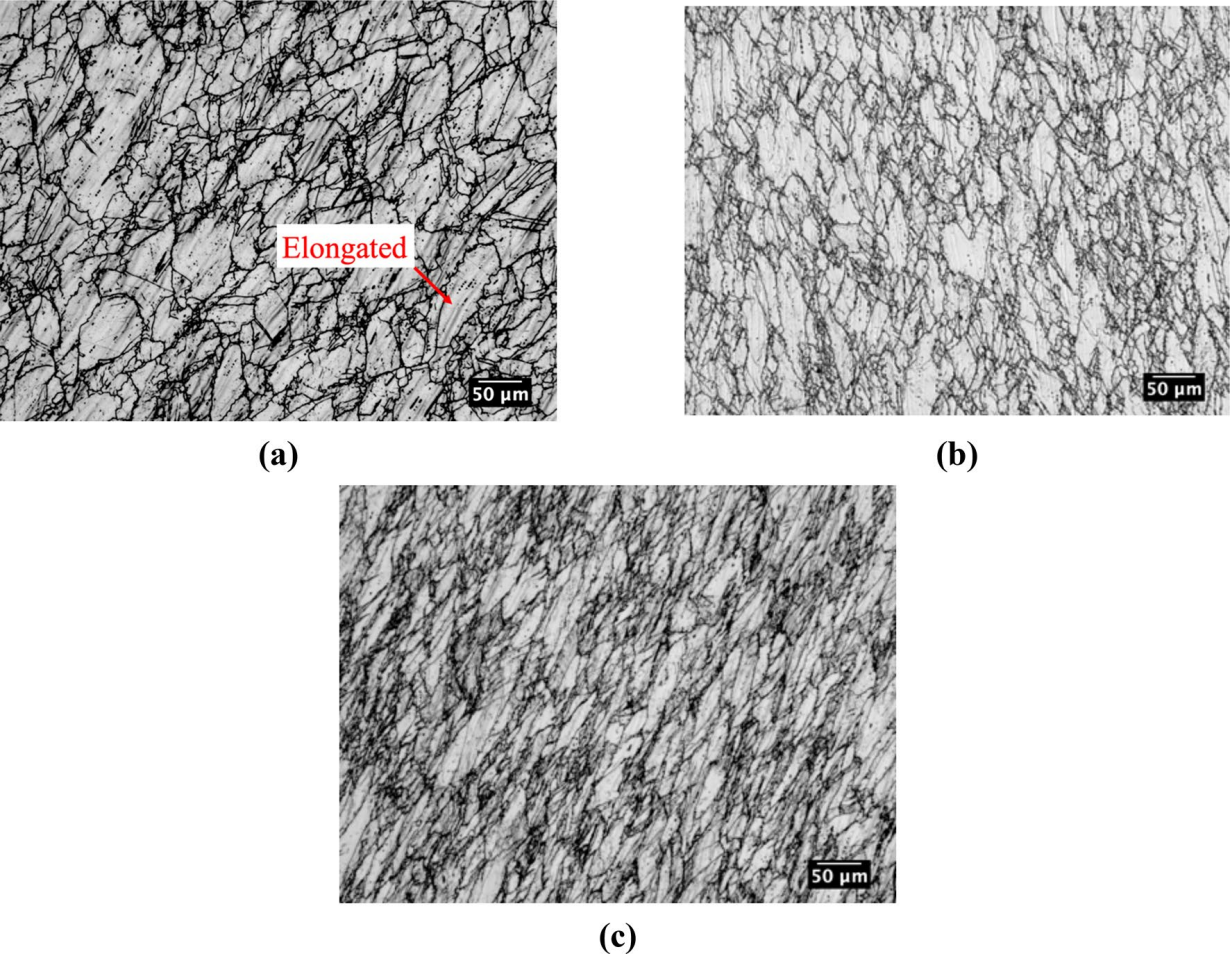
Microstructures generated using multi-step hot torsion (**a**) above T_nr_ at 1100 °C, (**b**) near T_nr_ at 1000 °C, and (**c**) below T_nr_ at 900 °C. Specimens were electrochemically etched in a solution of 60 pct nitric acid and 40 pct water and imaged at the effective radius along the torsional axis.

For multi-step hot torsion testing, microstructures above T_nr_ at 1100 °C, near T_nr_ at 1000 °C, and below T_nr_ at 900 °C are shown in Fig. 11a–c, respectively. Above T_nr_ at 1100 °C, the microstructure was predominantly recrystallized. However, it was evident that elongated grains, as identified in Fig. 11a, were present within the microstructure at this radial position suggesting that strain was retained in the austenite after the prior deformation steps at 1200 and 1150 °C. Near T_nr_ at 1000 °C, a partially recrystallized microstructure was observed, as shown in Fig. 11b by the presence of both elongated austenite grains and finer grains surrounding the elongated grains. The microstructure at 900 °C predominantly consisted of elongated grains. From the microstructures shown in Fig. 11, progressive grain refinement was observed as the number of deformation passes increased and deformation temperature decreased^20^. From above T_nr_ at 1100 °C to near T_nr_ at 1000 °C, the average grain size decreased from approximately 23–13 µm. As previously discussed, the progressive reduction in grain size was concluded to be the result of the additional deformation and recrystallization cycles inherent to multi-step hot torsion testing as opposed to double-hit compression testing where only a single deformation and recrystallization step occurs^2^.

**Figure 12:**
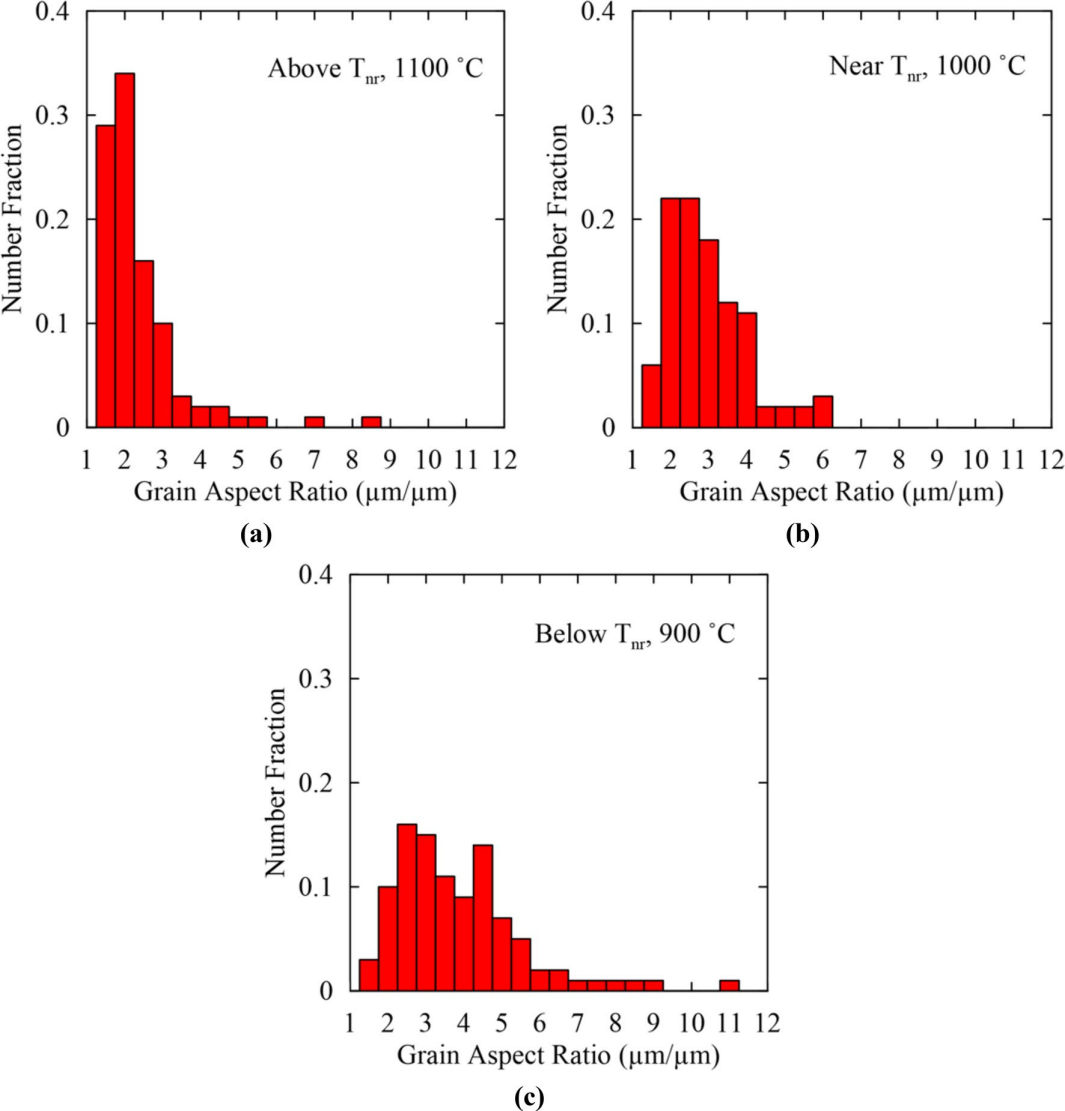
Grain aspect ratio histograms for multi-step hot torsion testing for temperatures (**a**) above T_nr_ at 1100 °C, (**b**) near T_nr_ at 1000 °C, and (**c**) below T_nr_ at 900 °C.

Changes in aspect ratio above T_nr_ at 1100 °C, near T_nr_ at 1000 °C, and below T_nr_ at 900 °C for the multi-step hot torsion test are shown in Fig. 12a–c, respectively. Above T_nr_, a fraction of 0.63 grains had aspect ratios between 1.0 and 2.0. This fraction was lower than for double-hit compression testing where a fraction of 0.74 grains had aspect ratios between 1.0 and 2.0. Multi-step hot torsion testing thus resulted in slightly greater strain accumulation above T_nr_ than double-hit compression, possibly due to the additional deformation and recrystallization cycles at 1200 and 1150 °C in the case of multi-step hot torsion testing. Evidence of strain accumulation was contrary to the expectation of nearly complete recrystallization above T_nr_. Near T_nr_ at 1000 °C, a fraction of 0.28 grains had aspect ratios between 1.0 and 2.0 with the remaining grains having higher aspect ratios. In comparison to double-hit compression testing, there was greater strain accumulation in the vicinity of T_nr_ for the multi-step hot torsion test. Below T_nr_, a fraction of 0.13 grains had aspect ratios between 1.0 and 2.0 indicating a predominantly deformed microstructure below T_nr_.

The broad distribution of aspect ratios in Fig. 12c suggests that grains may have varying degrees of strain accumulation, including grains that have been progressively deformed with no recrystallization (highest aspect ratios), and grains that have been recrystallized and subsequently deformed, indicated by grains with intermediate aspect ratios. From the comparatively low fraction of grains with high aspect ratios, *e.g.* above approximately 4.0 and 6.0 in Fig. 12b,c, respectively, it appeared that most grains underwent recrystallization at some point during the multi-step hot torsion test and then were subsequently deformed resulting in the largest fraction of grains having intermediate aspect ratios. However, the grains with the highest aspect ratios were believed to have been continually deformed between passes without recrystallization.

**Figure 13:**
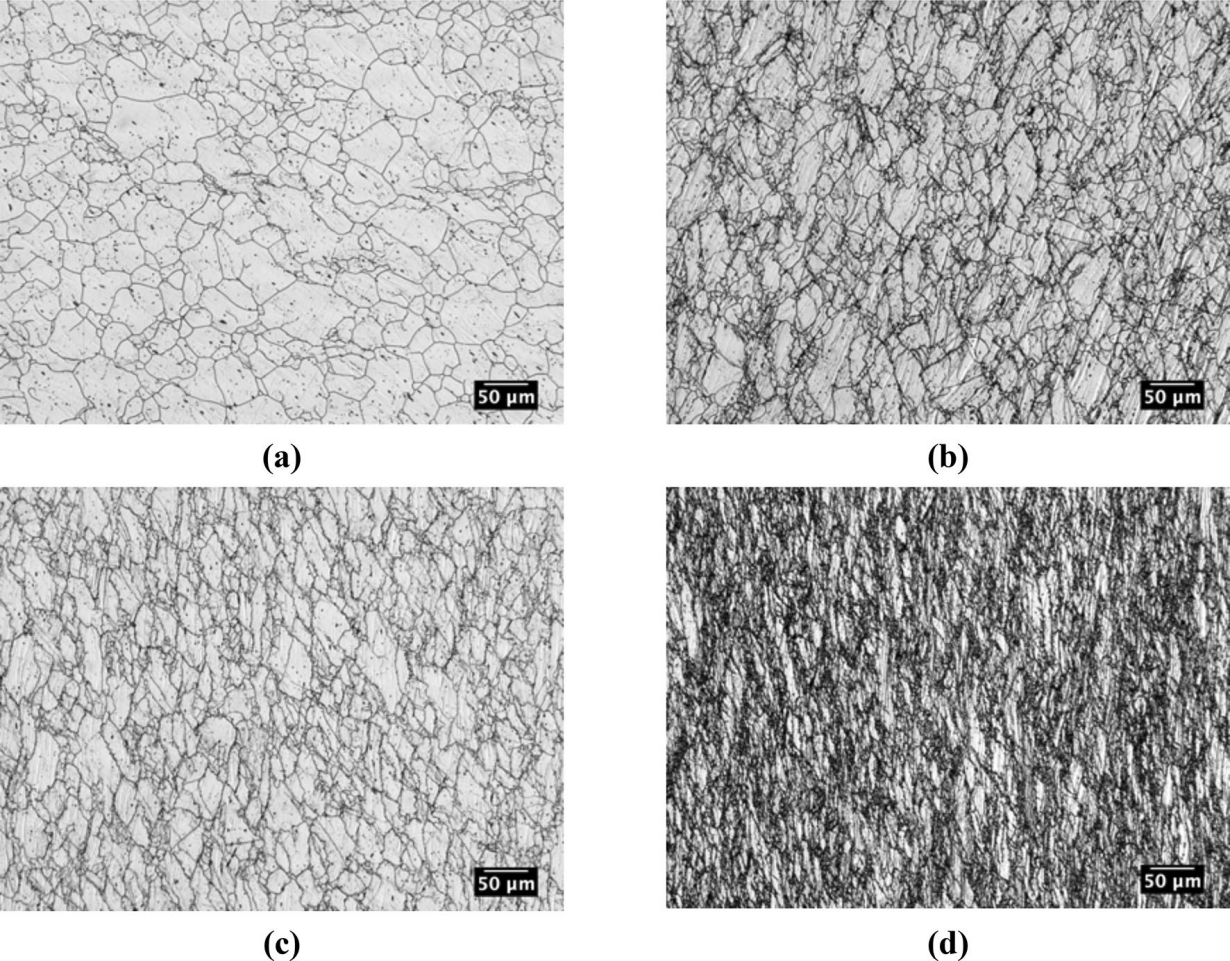
Microstructures generated with double-twist torsion (**a**) above T_nr_ at 1100 °C, (**b**) near T_nr_ at 1050 °C, (**c**) near T_nr_ at 1000 °C, and (**d**) below T_nr_ at 900 °C. Specimens were electrochemically etched with a solution of 60 pct nitric acid and 40 pct water and imaged at the effective radius along the torsional axis.

For the double-twist torsion test, the microstructures above T_nr_ at 1100 °C, near T_nr_ at 1050 °C, near T_nr_ at 1000 °C, and below T_nr_ at 900 °C are shown in Fig. 13a–d, respectively. For the double-twist torsion test, the average T_nr_ ranged between 1014 and 1041 °C, and both 1000 and 1050 °C were selected for microstructural analysis near T_nr_. Above T_nr_ at 1100 °C the microstructure appeared to consist primarily of recrystallized grains, consistent with the expectation of nearly complete static recrystallization between rolling passes above T_nr_. The microstructures near T_nr_ at 1050 °C and 1000 °C were similar in that they both contained a mixture of deformed and recrystallized grains. Similar to multi-step hot torsion testing, grain refinement was evident as the number of deformation and recrystallization cycles increased. Above T_nr_ at 1100 °C, the average grain size was approximately 26 µm. Upon reaching 1050 °C, the average grain size was reduced to approximately 12 µm. At 1000 °C, additional refinement of the austenite microstructure was minimal, with an average grain size of 11 µm. Below T_nr_, the austenite experienced extensive elongation of the grains, as shown in Fig. 13d. Some of the dark-etching indistinct regions in Fig. 13d are believed to consist of fine recrystallized grains surrounding the heavily deformed grains.

**Figure 14:**
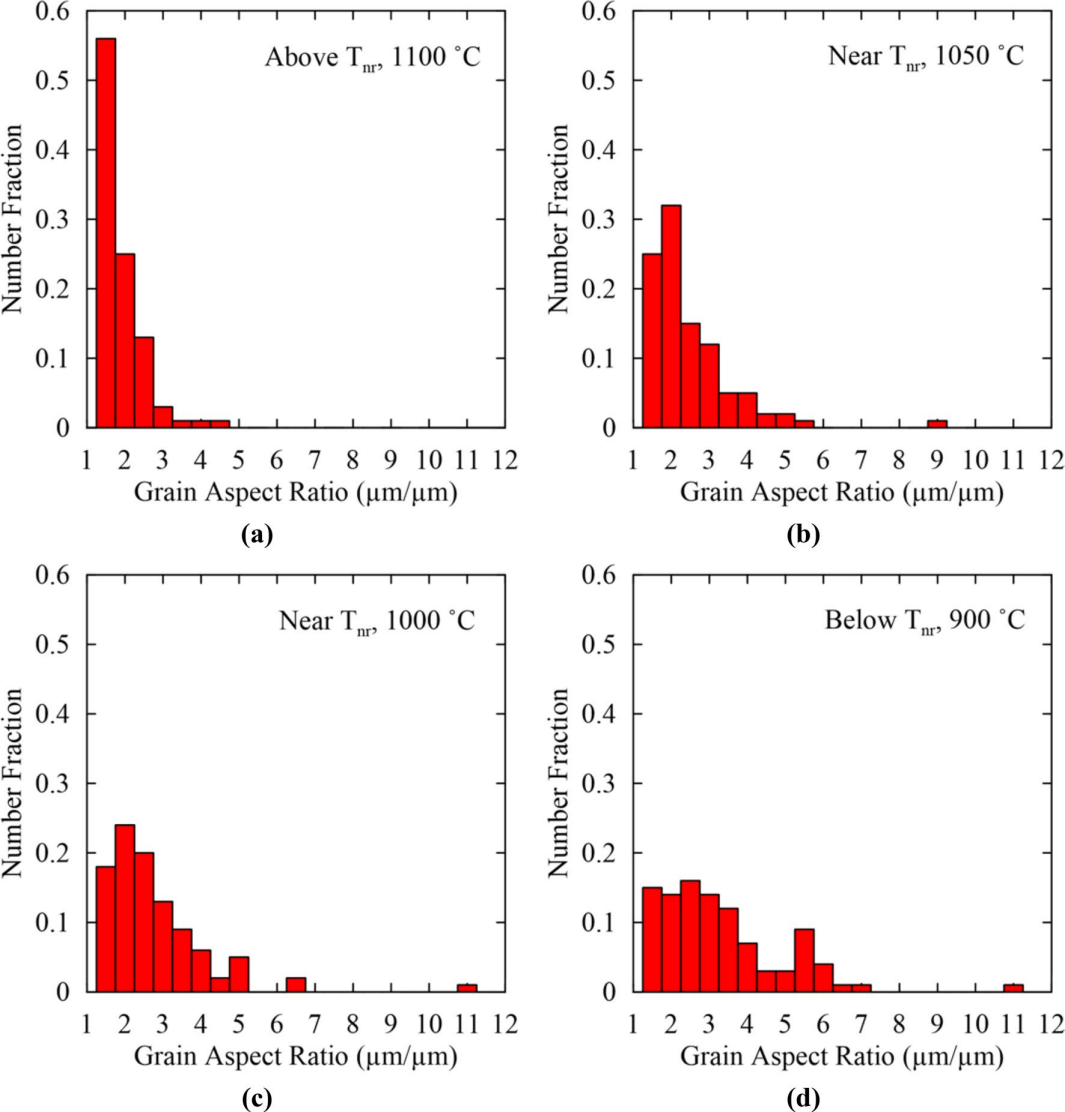
Grain aspect ratio histograms for double-twist torsion testing for temperatures above T_nr_ at (**a**) 1100 °C, near T_nr_ at (**b**) 1050 °C, near T_nr_ at (**c**) 1000 °C, and below T_nr_ at (**d**) 900 °C.

Similar to double-hit compression and multi-step hot torsion, strain accumulation was studied by examining changes in grain aspect ratio above T_nr_ at 1100 °C, near T_nr_ at 1050 and 1000 °C, and below T_nr_ at 900 °C as shown in Fig. 14a–d, respectively. Above T_nr_ at 1100 °C, a fraction of 0.81 grains had aspect ratios between 1.0 and 2.0 indicating a predominantly recrystallized microstructure, as expected above T_nr_. Near T_nr_ at 1050 °C, the fraction of grains with aspect ratios between 1.0 and 2.0 decreased, while grains having higher aspect ratios increased indicating a mixed microstructure. At 1000 °C, the fraction of grains having aspect ratios between 1.0 and 2.0 decreased further, indicating additional strain accumulation compared to 1050 °C. However, there was still a fraction (0.42) of grains with aspect ratios between 1.0 and 2.0, indicating partial recrystallization at 1000 °C. Below T_nr_ at 900 °C, the number fraction of grains with aspect ratios between 1.0 and 2.0 was 0.29 indicating further strain accumulation. As in the case of multi-step hot torsion, a broad distribution of aspect ratios was measured near and below T_nr_ and attributed to a combination of grains being continually deformed without recrystallization (high aspect ratios), grains recrystallized after deformation and deformed again (intermediate aspect ratios) and newly recrystallized grains (low aspect ratios).

**Figure 15:**
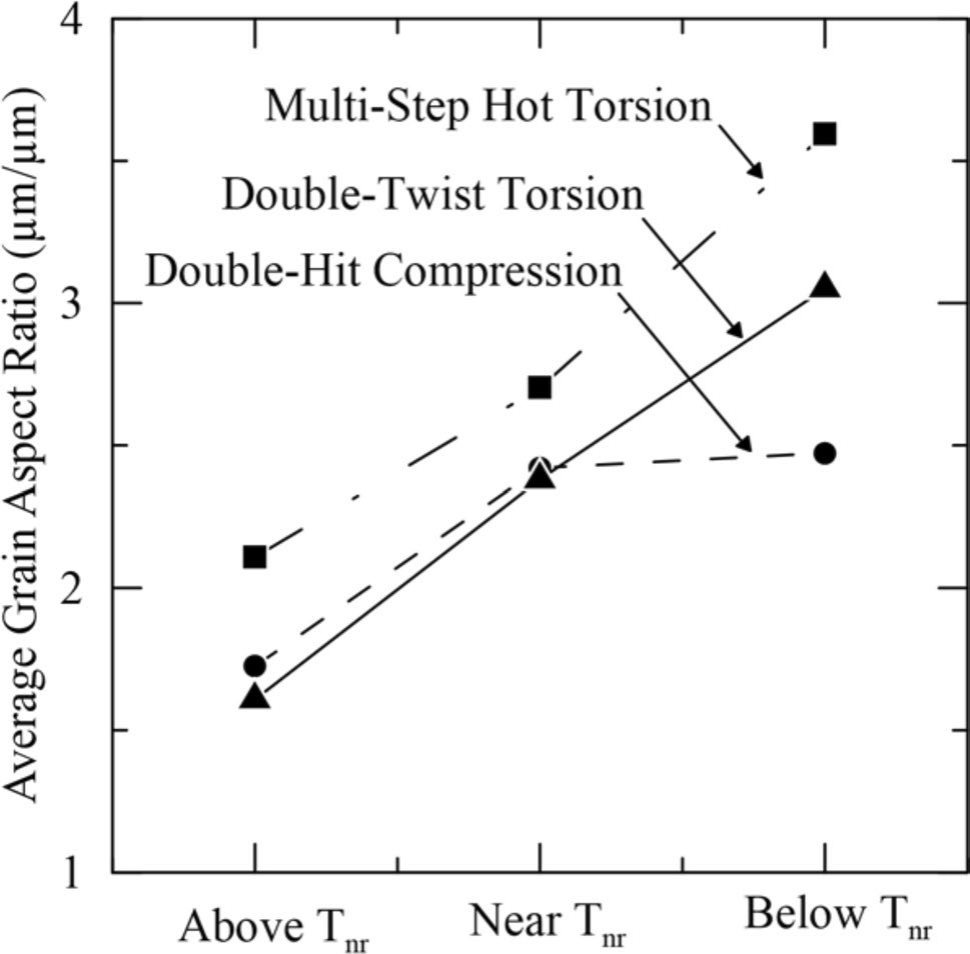
Average grain aspect ratios above, near, and below T_nr_ for double-hit compression, multi-step hot torsion, and double-twist torsion testing.

To better assess the effect of testing method on the extent of strain accumulation, the average grain aspect ratio was plotted above, near, and below T_nr_ for double-hit compression testing, multi-step hot torsion testing, and double-twist torsion testing in Fig. 15. Above T_nr_, both double-hit compression and double-twist torsion showed similar average grain aspect ratios slightly greater than 1.5. In the case of multi-step hot torsion, a higher average aspect ratio of 2.1 was observed. All three methods, therefore, resulted in predominantly recrystallized microstructures above T_nr_, though some strain accumulation was evident in the case of multi-step hot torsion testing. In the vicinity of T_nr_, all three methods showed similar average grain aspect ratios between 2.4 and 2.7. For double-twist torsion testing, the aspect ratios at 1050 and 1000 °C were averaged to calculate the average aspect ratio near T_nr_. With T_nr_ traditionally defined as the transition from complete to incomplete recrystallization, a predominantly recrystallized microstructure would be expected near T_nr_. All three methods, however, resulted in a mixed microstructure of recrystallized and deformed grains. It is understood that partial recrystallization of the austenite occurs over a temperature range^4,8^. Dutta and Sellars defined the recrystallization limit temperature (RLT) and recrystallization stop temperature (RST) as the upper and lower temperature limits over which partial recrystallization occurs, respectively^8^. The definition of T_nr_ as the transition from complete to incomplete recrystallization would closely correspond to the RLT, and a nearly recrystallized microstructure would be expected. In the case of all three testing methods, interpretation of the microstructure and measurement of average grain aspect ratio near the measured T_nr_ indicated that the behavior at T_nr_ represented a partially recrystallized microstructure lying within the temperature region of partial recrystallization bounded by the RST and RLT. T_nr_ defined as the transition from complete to incomplete recrystallization would be situated at slightly higher temperatures than the average T_nr_ values measured in the current study.

While the average grain aspect ratio was found to be similar for all three methods near the calculated T_nr_, considerable differences in grain size were observed, which may have influenced the recrystallization behavior. A higher T_nr_ of 1087 °C was measured for double-hit compression testing, and an average grain size of 30 µm was measured near the experimentally determined T_nr_. Multi-step hot torsion testing and double-twist torsion testing resulted in average T_nr_ values of 1037 and 1014–1041 °C, respectively, and both torsion testing methods had substantially smaller grain sizes near the experimentally determined T_nr_.

### Vickers microhardness

Vickers microhardness was used to assess the effect of the different processing routes on mechanical property evolution above, near, and below T_nr_. Microhardness values for each testing method and condition are shown in Table 3. For the double-hit compression test, the condition above T_nr_ showed the lowest hardness value, likely due to a combination of higher temperature and lower strain accumulation relative to the conditions near and below T_nr_. Similar hardness values were observed near and below T_nr_ for the double-hit compression test. The multi-step hot torsion test resulted in an increase in average hardness as the deformation temperature was reduced. The increase in hardness is likely the result of greater strain accumulation and microstructural refinement below T_nr_ in comparison to the double-hit compression test. For the double-twist torsion test, similar hardness values were observed above T_nr_ at 1100 °C and near T_nr_ at 1050 °C. However, the hardness increased both at 1000 and 900 °C, showing a similar trend as multi-step hot torsion testing. Both torsion testing methods resulted in a higher hardness compared to the double-hit compression test for all testing conditions, likely due to the lack of microstructural refinement observed in the double-hit compression test. Below T_nr_ at 900 °C, the double-twist torsion test resulted in a higher average hardness than the multi-step hot torsion test, which can be expected from the greater microstructural refinement observed in the case of the double-twist torsion test compared to the multi-step hot torsion test at 900 °C.

**Table 3:** Vickers microhardness evolution during thermomechanical processing.

Method	Temperature (˚C)	Hardness (HV)
Double-hit compression	1150 (above T_nr_)	185 ± 6
	1100 (near T_nr_)	199 ± 3
	1050 (below T_nr_)	201 ± 4
Multi-step hot torsion	1100 (above T_nr_)	220 ± 3
	1000 (near T_nr_)	226 ± 6
	900 (below T_nr_)	270 ± 7
Double-twist torsion	1100 (above T_nr_)	211 ± 8
	1050 (near T_nr_)	209 ± 5
	1000 (near T_nr_)	242 ± 6
	900 (below T_nr_)	282 ± 8

## Conclusions

A double-twist torsion test has been developed as an alternative method for determining T_nr_ with advantages over the traditional methods of double-hit compression and multi-step hot torsion testing. From the double-twist torsion test, T_nr_ can be determined from measurements of fractional softening, enabling the extent of recrystallization during thermomechanical processing to be inferred. Because the test is conducted in torsion, effects of multiple deformation and recrystallization cycles on the experimental T_nr_ are incorporated, unlike double-hit compression testing where a single specimen is typically used to simulate rolling at a single temperature. The double-twist torsion test was developed and compared to traditional methods using an austenitic 316 stainless steel where differences in the austenite microstructure above, near, and below the experimental T_nr_ could be directly assessed. Comparing the measured T_nr_ values and associated microstructures determined with double-hit compression, multi-step hot torsion, and double-twist torsion testing the following conclusions may be drawn.

- The double-twist torsion test resulted in measured T_nr_ values similar to those determined with multi-step hot torsion and lower than those determined with double-hit compression testing. The effect of data analysis method (i.e. fractional softening or MFS versus inverse temperature approach) was found to have a modest effect on T_nr_ in the case of double-twist torsion testing while a larger difference in T_nr_ was calculated between double-hit compression and multi-step hot torsion testing.
- The measured T_nr_ lies within the temperature region of partial recrystallization. All three testing methods resulted in a partially recrystallized microstructure in the vicinity of the measured T_nr_, contrary to the definition of T_nr_ as the transition from complete to incomplete recrystallization (wherein the microstructure would be predominantly recrystallized around T_nr_). T_nr_, according to the traditional definition, would lie at a higher temperature than T_nr_ measured with any of the three methods.
- Grain size varied considerably near the calculated T_nr_. The lower T_nr_ values associated with both multi-step and double-twist torsion testing corresponded to a microstructure with a finer average grain size compared to double-hit compression testing, which predicted a higher T_nr_ and resulted in a corresponding microstructure with a larger grain size. The present study appears to confirm the hypothesis that increasing grain boundary area reduces T_nr_. However, microstructures after torsion testing have only been analyzed thus far at the effective radius of the torsion specimens. The variation in imparted strain through the cross section of a torsion specimen and its effect on fractional softening measurements and the extent of recrystallization may also contribute to the differences in T_nr_ predicted by double-hit compression and torsion testing methods.
- The average austenite grain aspect ratio near the measured T_nr_ did not vary substantially with testing method. All three methods showed similar average grain aspect ratios near T_nr_ indicating similar levels of strain accumulation in the austenite despite widely varying T_nr_ values.
- Both torsion testing methods resulted in higher hardness values above, near and below T_nr_ compared to double-hit compression, likely due to the lack of microstructural refinement in the case of double-hit compression testing. The double-twist torsion test resulted in a slightly higher average hardness below T_nr_ than the multi-step hot torsion test, possibly due to the additional imparted strain and greater microstructural refinement in the case of the double-twist torsion test.

Because the extent of recrystallization can be inferred throughout a thermomechanical processing schedule with measurements of fractional softening, the double-twist torsion test developed here may best capture the fundamental nature of T_nr_ as a microstructural phenomenon and the occurrence of partial recrystallization over a range of temperatures. Double-hit compression testing is also capable of linking the extent of austenite recrystallization to changes in mechanical data through measurements of fractional softening. However, it does not incorporate effects of multiple deformation and recrystallization steps. Multi-step hot torsion testing incorporates multiple deformation and recrystallization steps, but direct microstructural observation is needed to characterize the extent of recrystallization. The double-twist torsion test incorporates multiple deformation and recrystallization cycles and allows changes in austenite recrystallization to be predicted through fractional softening measurements.

## Data Availability

The datasets generated during and/or analyzed during the current study are available from the corresponding author on reasonable request.
